# Natural Products as a Tool to Modulate the Activity and Expression of Multidrug Resistance Proteins of Intestinal Barrier

**DOI:** 10.3390/jox13020014

**Published:** 2023-03-25

**Authors:** Carlos Martins-Gomes, Amélia M. Silva

**Affiliations:** 1 Centre for Research and Technology of Agro-Environmental and Biological Sciences (CITAB), University of Trás-os-Montes and Alto Douro (UTAD), 5001-801 Vila Real, Portugal; 2 Department of Biology and Environment, School of Life Sciences and Environment, University of Trás-os-Montes and Alto Douro (UTAD), 5001-801 Vila Real, Portugal

**Keywords:** multidrug-resistant proteins, P-glycoprotein, MRP1, ABCG2, phytochemicals, aromatic and medicinal plants, nutraceuticals, intestinal barrier, colorectal cancer, xenobiotic transport

## Abstract

The role of intestinal barrier homeostasis in an individual’s general well-being has been widely addressed by the scientific community. Colorectal cancer is among the illnesses that most affect this biological barrier. While chemotherapy is the first choice to treat this type of cancer, multidrug resistance (MDR) is the major setback against the commonly used drugs, with the ATP-binding cassette transporters (ABC transporters) being the major players. The role of P-glycoprotein (P-gp), multidrug resistance protein 1 (MRP1), or breast cancer resistance protein (ABCG2) in the efflux of chemotherapeutic drugs is well described in cancer cells, highlighting these proteins as interesting druggable targets to reverse MDR, decrease drug dosage, and consequently undesired toxicity. Natural products, especially phytochemicals, have a wide diversity of chemical structures, and some particular classes, such as phenolic acids, flavonoids, or pentacyclic triterpenoids, have been reported as inhibitors of P-gp, MRP1, and ABCG2, being able to sensitize cancer cells to chemotherapy drugs. Nevertheless, ABC transporters play a vital role in the cell’s defense against xenobiotics, and some phytochemicals have also been shown to induce the transporters’ activity. A balance must be obtained between xenobiotic efflux in non-tumor cells and bioaccumulation of chemotherapy drugs in cancer cells, in which ABC transporters are essential and natural products play a pivotal role that must be further analyzed. This review summarizes the knowledge concerning the nomenclature and function of ABC-transporters, emphasizing their role in the intestinal barrier cells. In addition, it also focuses on the role of natural products commonly found in food products, e.g., phytochemicals, as modulators of ABC-transporter activity and expression, which are promising nutraceutical molecules to formulate new drug combinations to overcome multidrug resistance.

## 1. Introduction

Biological barriers are essential to maintaining organisms’ homeostasis by being able to protect against external aggressors that may arise as microorganisms, xenobiotics, or physical environmental agents (e.g., UV radiation). These barriers are equipped with various defense mechanisms capable of withstanding and countering external aggressions while also maintaining vital processes such as nutrient absorption [1,2]. Equally fundamental to maintaining homeostasis and basic life functions is the ability of a barrier cell to move molecules, when necessary, a process accomplished by membrane channels and transporters that regulate the flow of ions, water, glucose, or even larger molecules. Some classes of these transporters use the energy of ATP (adenosine triphosphate) to perform the active transport of molecules, among which the ABC (ATP-binding cassette) transporters stand out. These transporters are responsible for the transport of a wide variety of substrates, such as xenobiotics, amino acids, lipids, polysaccharides, ions, and others, and while some transporters have well-defined substrates, others may transport various types of molecules [3,4]. Their expressions and import/export activities are aligned according to each cell type for functions such as the efflux of phospholipids, cholesterol, and other metabolism products [3,4], and they are also actively involved in xenobiotic efflux [4,5,6]. Nevertheless, the biochemical processes involved in the response to xenobiotics can also act against the organism, especially when the objective is to deliver a drug to a specific target, such as tumor cells, which frequently use xenobiotic defense mechanisms to evade anti-tumor drug activity. The resistance of cancer cells to various cytotoxic drugs used in chemotherapy is a widely known and studied process that embraces genetic factors and their enhanced ability to repair DNA, metabolize xenobiotics, and produce alterations in the plasmatic membrane aiming to reduce the cytoplasmic accumulation of drugs and increase drug efflux [7,8]. These are often subsumed under the well-known concept of multidrug resistance (MDR), which represents the resistance of cancer cells to drugs that can be pre-existent or acquired, preventing the prolonged and efficient use of chemotherapeutic drugs, in which ABC transporters play a critical role [9].

The different expression of members of this large family of transporters has been linked to the poor prognosis and poor outcome of certain cancer types; it is estimated that multidrug resistance accounts for 90% of cancer-related deaths in patients undergoing chemotherapy [1,7,10,11]. The first report on MDR refers to a colchicine-resistant clone of CHO (Chinese hamster ovary) cells [12], which contributed to the identification of a cell’s surface glycoprotein, identified by the authors as P-glycoprotein (P-gp) [13]. Later studies, using similar cell lines, also reported resistance to drugs such as daunorubicin, vinblastine, or taxol [14,15]. Since then, the physiological role of ABC transporters both in health and in disease has been the target of countless studies. Therefore, these transporters present an interesting druggable target in which natural products, with a wide variety of chemical structures, provide a large spectral range of possibilities to counter multidrug resistance. In this review, we provide an approach to multidrug resistance modulation by various phytochemical classes, with emphasis on their role in modulating certain ABC transporters present at the intestinal barrier.

## 2. ATP Binding Cassette (ABC) Transporters Family

Distributed in all organisms, ABC transporters are active transporters that comprise a high number of transmembrane proteins with a common base structure and the need to hydrolyze ATP to achieve active transport. Prokaryotes use these transporters for the influx of hydrophilic molecules, while both prokaryotes and eukaryotes use ABC transporters to perform the transport of various molecules, such as lipids, proteins, and various xenobiotics [5,16]. In addition to ABC transporters at plasmatic membranes, eukaryotes can also present ABC transporters in organelle membranes [17]. Chemotherapeutic drugs, often large hydrophobic molecules, are common substrates of these transporters [5]. In addition to tumor resistance, ABC transporters also play a role in cystic fibrosis, degeneration of the retina, and defective lipid metabolism [18].

Concerning the molecular structure, ABC transporters present the nucleotide binding domains (commonly referred to as NBDs), which consist of the portion responsible for ATP binding and hydrolysis, with well-conserved sequences, the unifying characteristic of ABC systems, and the transmembrane domains (TMDs), which are more variable [16,17]. While in prokaryotes the functional unit is generally constituted by a homo- or heterodimer, with each monomer composed of a NBD and a TMD (half-transporter), in eukaryotes the functional unit often consists of a monomer comprising two NBDs and two TMDs [19]. These four domains (two NBDs and two TMDs) are commonly encoded by a single gene (full-size transporter) or by two genes resulting in two half-transporters (half-size transporter; with one NBD and one TMD) that then assemble into a functional transporter [18].

The classification of ABC transporters is based either on their function, the classical characterization, or based on the classification of the genes encoding the transporter, which is currently the preferred one. In addition, a new proposal for ABC transporters’ classification is also under analysis, based on the TMD fold, resulting from increased knowledge on the transporters structure due to improved single-particle cryo-electron microscopy and X-ray crystallography methodologies [20]. Regarding the classical characterization, based on TMD sequence homology, seven types of ABC transporters are recognized, according to the direction of substrate translocation, of which three are importers (types I, II, and III), thus responsible for the movement of molecules from the extracellular to the intracellular space, with higher relevance in prokaryotes. As ABC importers have recently been identified in some algae and non-vascular plants but not in other eukaryotes, they will not be addressed in this review; two are exporters (types IV and V), which have been found in all phyla, and the other two are extruders or mechanotransducers (types VI and VII) and will not be discussed here either [6,18,21]. As some ABC transporters classified in class IV were shown to have functions different from exporting molecules, the classification based on substrate movement direction is being avoided [6,18].

**Figure 1:**
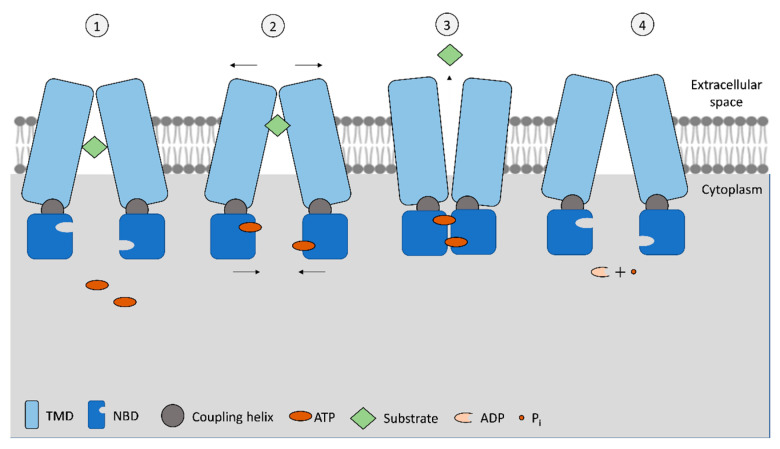
Schematization of the basic mechanism of ABC exporters. In the standard inward-facing phase (1), open NBD conformation, the transporter allows for the substrate to move to the translocation pore from the cytoplasm (or from the membrane bilayer), and then, upon ATP binding to NBSs (2), a conformational change is initiated to the outward-facing position. As NBD monomers approach each other and connect, while TMDs pull away, this is translated by the coupling helixes on the TMDs, allowing the release of the substrate to the extracellular space (3). Subsequently, ATP is hydrolyzed, and once hydrolyzed into ADP + P_i,_ the transporter acquires the original inward-facing conformation (4). Figure adapted from [17,18].

The ABC exporters, types IV and V, in eukaryotes are frequently composed of a polypeptide chain that comprises all domains. The NBDs are ATPases that bind and hydrolyze ATP, presenting an ATP-binding site in each NBD (Figure 1). The basic mechanism of drug efflux by ABC exporters is illustrated in Figure 1.

Nevertheless, the use of two ATP molecules to transport each substrate molecule or if both ATPs are simultaneously hydrolyzed is not consensual, as it has been observed that some ABC transporters require two ATP molecules while others have only one of the NBDs capable of hydrolyzing ATP [17]. When the binding to substrate occurs, NBDs dimerize, and only then can ATP be hydrolyzed. After ATP hydrolysis, ADP and a phosphate ion (P_i_) are released, the NBD dimer loses stability, and NBD monomers separate (Figure 1). The first step allows the transference of energy from ATP molecules to TMDs, promoting the transport of their substrate. In addition to NBDs and TMDs, a coupling helix has been identified in exporter-type ABCs. This helix is located in the cytoplasmic end of TMD monomer and connects with NBD monomer, being part of the transference of conformational energy between the two domains [17,21]. Unlike ABC importers, which have more defined substrates and binding sites, the wide variety of ABC exporters also present the ability to bind molecules of variable size and chemical structure [17]. Even within ABC exporters, members of type IV transporters present a wider range of substrates than type V transporters, which can be attributed to different TMDs’ conformation that allow the accommodation of amphiphilic, hydrophilic, and hydrophobic substrates, a key differentiation for their role as multidrug transporters [4]. Even more, different substrate binding can occur depending on whether the substrate efflux is carried from the cytoplasm or from within the membrane lipid bilayer, depending on the chemical nature of the substrate [17]. 

**Figure 2:**
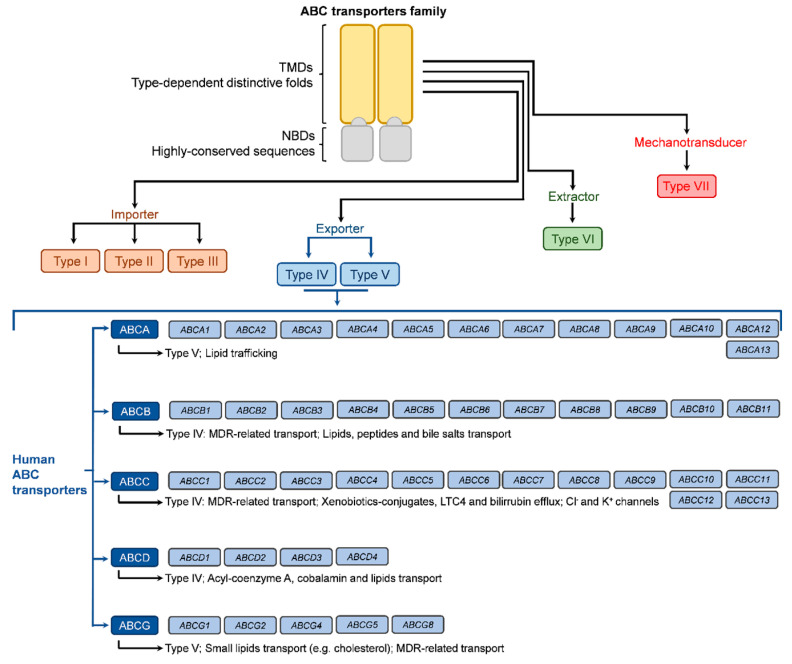
Illustration of overall ABC transporters by type, according to TMD conformation, and of human ABC transporters according to the genes’ nomenclature.

As stated above and illustrated in Figure 2, the ABC transporter nomenclature based on the encoding gene is increasingly accepted and recommended. The human genome carries at least 48 *ABC* genes, with some publications indicating 49 genes, which are arranged in seven subfamilies (A to G; based on the sequence homology of nucleotide binding domains) and code for the full-transporters or half-transporters (four genes) of the ABC transporters superfamily [22,23], thus not corresponding to 49 ABC full transporters. These transporters regulate the plasmatic membrane as well as the traffic through organelles’ membranes, including the Golgi apparatus, endoplasmic reticulum, and mitochondria. Their structure and sequence homology allowed the division into seven subfamilies named from ABCA to ABCG, according to the encoding genes [16,17,22]. Nevertheless, the classification includes members that are not transporters, as is the case with subfamily ABCE (one member coded by the *ABCE* gene) and subfamily ABCF (four members coded by genes *ABCF1* to *ABCF4*). Both of these subfamilies are involved in ribosomal remodeling and mRNA translation [24]. The ABCA subfamily is comprised of 12 members (coded by genes *ABCA1* to *ABCA10*, *ABCA12,* and *ABCA13*), classified as type V regarding their TMD conformation and presenting large extracellular domains (ECD) as a differentiating factor. Their major role is in lipid trafficking [4,22]. The four members of the ABCD subfamily (type IV transporters coded by genes *ABCD1* to *ABCD4*) are also involved in lipid transport but also specialize in the efflux of acyl-coenzyme esters [4]. Like the ABCA subfamily, these present a lower level of relevance regarding MDR.

Among ABC transporters, P-glycoprotein (P-gp), multidrug resistance-associated protein 1 (MRP1 or ABCC1), and ATP-binding cassette subfamily G member 2 (ABCG2) have been studied for their implication in MDR, with great relevance for colorectal cancer [25]. Equally relevant is the ability of certain phytochemicals commonly found in the human diet to interact with these transporters and prevent the efflux of chemotherapeutic drugs. This bioactivity has been described for various herbal extracts, isolated phytochemicals, and other natural compounds, although some of them may act as inhibitors, inducers, or substrates of ABC transporters. Therefore, this topic requires a comprehensive assessment of the phytochemical-produced modulatory effect [25,26].

The advances in analytical methods used to evaluate these transporters’ structure and function, particularly in ABC exporters, namely by cryogenic electron microscopy, allowed for the determination of their conformation with the various ligands for each receptor [4]. Among the conformations confirmed for these exporters, the occurrence of transporters in occluded, outward-facing, inward-facing, lateral-opening, quatrefoil, or propeller conformations [4] was reported. Due to the high variability of TMDs conformation, the mechanism behind exporters’ activity is still being elucidated, with variations from the basic functionality presented in Figure 1. Currently, the main doubts regarding ABC transporters’ mechanism are related to ATP binding to NBDs, substrate binding to TMDs, and the order of events. For example, it is hypothesized that substrate-TMD binding may increase the binding of ATP to NBD. Additionally, the symmetry/asymmetry in ATP hydrolysis by NBDs is still being discussed, as the transference of energy from one or two ATP molecules is not clarified [27]. In addition to the classic switch model depicted in Figure 1, other options, such as the reciprocating model, have been proposed. In this model, the TMD dimer would contain two substrate-transmembrane translocation channels, each paired with a nucleotide binding site (in the NBDs) [28]. In one pair, the substrate and ATP bind to their respective high affinity sites in TMDs and NBDs simultaneously, in an inward-facing conformation. The substrate is then occluded on the inner side of the plasmatic membrane, and while the ATP in the nucleotide binding site is hydrolyzed, the substrate is moved to the outer side of the membrane. The release of ADP from the nucleotide binding site is accompanied by the release of the substrate from a low-affinity binding site on the extracellular side. In this model, the cycle of each pair of substrate-binding sites/ATP-binding sites in a full transporter operates out of phase [28]. Nevertheless, as described above, as new findings are reported not only for exporters but for all ABC transporters, the knowledge regarding currently accepted mechanisms is always evolving.

### 2.1. Main ABC-Transporters Implicated in Multi-Drug Resistance in Colorectal Cancer

As stated above, the intestinal barrier is equipped with certain ABC-transporters that actively contribute to xenobiotic extrusion and also have a relevant role in colorectal cancer resistance to chemotherapy drugs, as drugs used in chemotherapy are substrates for these transporters, ultimately compromising the success of chemotherapy. Lately, many articles have reported the role of natural products, in particular phytochemicals, in modulating the activity and expression of these transporters, constituting therefore a set of molecules with promising activity to overcome or reduce, MDR. The role of phytochemicals as modulators of the main ABC-transporters of intestinal barrier cells, as well as their specific activities, is presented and discussed below.

#### 2.1.1. P-glycoprotein

With ubiquitous distribution through the various tissues and the capacity to export different substrates, P-gp, also known as MDR1 (multidrug resistance protein 1) or ABCB1 (ATP-binding cassette sub-family B member 1), is highly addressed for its role in MDR. Regarding physiological barriers, P-gp plays a crucial role in the homeostasis of both the intestinal barrier and the blood-brain barrier [1,18]. Although both NBD domains present active ATP hydrolysis binding sites, they function asymmetrically, suggesting asymmetry of the inward-facing structure. TMDs present polyspecificity due to their great range of inward-facing conformations [1,18]. This 170 kDa transmembrane protein is coded by the *ABCB1*/*MDR1* gene, and one of its intended physiological functions at the intestinal barrier is toxin clearance, preventing its systemic absorption [1]. Its expression varies along the intestinal tract, with higher expression in the colon, followed by the jejunum and ileum [7]. This transporter can operate with a long list of substrates such as steroid compounds, protease inhibitors, immunosuppressor drugs, cardiovascular drugs, antibiotics, antihistamines, and one of the most concerning groups, chemotherapy drugs such as doxorubicin [1]. Precisely in colorectal cancer, the inhibition of P-gp has been seen as an opportunity to reverse MDR, even more so when it is described that this type of cancer has high P-gp expression [7]. However, in normal physiological conditions, P-gp limits xenobiotic absorption, preventing the bioaccumulation of toxic compounds [29].

Various phytochemicals have been shown to interact with P-gp. For example, resveratrol’s action as an anti-proliferative agent in colorectal cancer cells is dependent on P-gp activity, which transports resveratrol as a xenobiotic and thus prevents its action [8]. Other compounds, such as chrysin, induce P-gp activity, while kaempferol, baicalein, galabridin, and neostenine are also substrates for P-gp [25,26,30].

#### 2.1.2. Multidrug Resistance-Associated Proteins Subfamily

The last decade of the 20th century has brought brand new insights into the MRP subfamily, when its most addressed member, the multidrug resistance-associated protein 1 (MRP1), had its gene, *ABCC1*, cloned in 1992 [31], to which followed proteins MRP2 (also known as cMOAT) to MRP6 from 1996 to 1998, coded by genes *ABCC2* to *ABCC6*, respectively [32]. The expression of MRP in various tissues varies depending on the MRP member. While MRP1 and MRP5 are generally expressed in various tissues, MRP2, MRP3, MRP4, and MRP6 have higher expression in the liver and kidney [32]. In addition, MRP2 has high expression in the gut and MRP3 in the adrenals and pancreas [32]. MRP4 is the member with the more exclusive expression, being mainly present in the prostate, lung, muscle, pancreas, bladder, ovary, and testis [32]. The addition of MRP7 (*ABCC10* gene) to the MRP subfamily was reported in 2001 [33], to which was also later added MRP8 (*ABCC11* gene) and MRP9 (*ABCC12* gene); these last two are greatly expressed in breast cancer [34,35]. The MDR subfamily also includes proteins coded by genes *ABCC7*, *ABCC8,* and *ABCC9*, although these are not involved in drug efflux. The nine MRP members are structurally divided into two groups: (i) MRP 4, 5, 8, and 9, the shorter MRPs with two membrane-spanning domains, and (ii) the longer MRPs, with an extra membrane-spanning domain, comprising MRP 1, 2, 3, 6, and 7 [36].

MRP1 is the most relevant member when it comes to MDR. Despite its generalized expression, the lung, skin, intestine, and kidney are some of the tissues with higher expression, while the liver presents lower expression [37]. When compared to P-gp, the MRP1 sequence is only 15% similar and presents a different structure since, as mentioned above, MRP1 presents an extra membrane-spanning domain with five transmembrane helices. Among the substrates of this transporter are chemotherapy drugs such as anthracyclines (e.g., doxorubicin), vincristine, camptothecin, or etoposide, but unlike P-gp, it is not involved in the resistance to taxanes (e.g., taxol) [38,39]. Another distinction between MRP1 and P-gp is localization. While P-gp and MRP2 are found in the apical membranes of polarized intestinal epithelial cells, MRP1 is located in the basolateral membrane [39]. The normal physiological functions of this transporter involve the efflux of xenobiotics or their glutathione, glucoronate, or sulfate conjugates, which are involved in the cellular extrusion of xenobiotics after phase II, which is why they are also referred to as GS-X pumps (GS for glutathione conjugates and X for the xenobiotic). Glutathione involvement in MRP1 transport is further extended, as it may be necessary to efflux some substrates in a co-transport mechanism. Even more, oxidized glutathione (GSSG) also acts as a substrate for MRP1, a mechanism thought to be part of the system to maintain low intracellular GSSG concentrations and thus to promote redox balance under oxidative events [37,38]. Sulfated bile salts, atorvastatin, flutamide, folic acid, or bilirubin are also substrates of this transporter, which also plays a role in inflammatory pathways through the transport of leukotriene C4 (LTC4) in mast cells [37,38,39].

MRP2 is also involved in xenobiotic efflux; MRP3 transports metabolism conjugates with a higher affinity for glucoronates instead of glutathione conjugates such as MRP1 [39]. Concerning MRP1 inhibition, verapamil (often used as a positive control in MDR reversion studies) is an inhibitor of both P-gp and MRP1 [39]. Some natural compounds have also been described as MRP1 inhibitors, such as the flavonoids quercetin and genistein [39]. However, natural compounds, especially mixtures such as herbal extracts, may show contradictory actions, as other flavonoids (apigenin, kaempferol, or naringenin) have been shown to stimulate MRP1 or be co-transported [39]. As discussed generally for ABC transporters, the inhibition of MRP1 as a MDR-inducing transporter is highly addressed, as the expression of this transporter has been confirmed in various tumor types, mostly of the solid type, and with implications for disease outcome [37]. Specifically in colorectal cancer, MRP1 has been pointed out as a therapeutic target to reverse MDR, as the transporter activity reduces the action of chemotherapeutic drugs, reducing apoptosis induction [40].

It is worth noting that not all genes in this family code for exporters, as is the case with *ABCC7*, which codes for the cystic fibrosis transmembrane conductance regulator, an ATP-gated chloride channel, and *ABCC8*/*ABCC9*, which code for ATP-gated potassium channels [20,27].

#### 2.1.3. Subfamily G

Among the members of the ABCG subfamily, some have defined roles in certain cell types, with less relevance to MDR. For example, ABCG1 regulates lipid homeostasis and is responsible for cholesterol and phospholipid transport in macrophages, a key aspect of their role in the inflammatory cascade, while ABCG5 and ABCG8 are sterol transporters expressed in the liver and intestinal tract [41,42,43]. In the case of ABCG1, it was found to be overexpressed in osteosarcoma cells, accompanied by increased expression of P-gp, and with an active role in the resistance to doxorubicin and etoposide [44]. ABCG1 overexpression was also observed in cells mediating the inflammatory process in diabetic rats’ small intestine [45]. Nevertheless, when considering MDR, ABCG2 (ATP-binding cassette superfamily G member 2) is the most remarkable member for its high expression in cancer cells and in cancer stem cells [41,42]. This protein also presents a wide variety of substrates, contributing to drug efflux in MDR, which is why it is also referred to as breast cancer resistance protein (BCRP) [41,42]. ABCG2 is a half-transporter, with one NBD and one TMD, and therefore it is possible that the transporter function is carried out as a homodimer or as oligomers, since ABC transporters require two NBDs and two TMDs [41,42,46]. Under normal physiological conditions, ABCG2 is expressed in the liver and regulates the efflux of xenobiotics in their sulfate or glucuronide conjugated form after phase-II hepatic metabolism [41]. At intestinal level, the duodenum has the highest expression of this transporter, which decreases towards the colon [42]. Regarding MDR, the ABCG2 transporter has been shown to contribute to the efflux of various drugs, including doxorubicin, daunorubicin, 5-fluorouracil, topoisomerase inhibitors (e.g., 9-aminocamptothecin, mitoxantrone, and etoposide), sorafenib, and diclofenac, but it also interacts with phytochemicals, such as flavonoids [41,42]. In fact, Tan, et al. 2013 [47] tested the interaction of 56 compounds of various classes of phytochemicals with ABCG2 and confirmed the inhibition of ABCG2 activity by compounds such as quercetin, chrysoeriol, ursolic acid, oleanolic acid, sinapic acid, ellagic acid, and berberine, while reporting a lower inhibitory effect by phenolic acids when compared to the flavonoids or triterpenes [47].

In addition to the ABC transporters mentioned above, other members of this family are key intervenients in intestinal barrier functions and homeostasis, although they present a less significant role in MDR. This is the case of ABCA1, a transporter first cloned in 1994 with two TMDs, two NBDs, and two extracellular domains, involved mainly in lipid metabolism, where it regulates cholesterol efflux and high-density lipoprotein (HDL) biogenesis [48,49,50]. This transporter regulates cholesterol efflux to form HDL, while the mentioned above ABCG1 regulates efflux to mature HDL, these two transporters are responsible for at least 60% of the cholesterol/HDL pathway in macrophages [43]. In addition, ABCA1 is linked to an anti-inflammatory response, as decreased ABCA1 expression in macrophages leads to increased cholesterol in membrane rafts, which potentiates the signal transduction induced by LPS (lipopolysaccharides) in TLR4 (toll-like receptor 4), as well as participating in STAT3 (signal transducer and activator of transcription 3) pathway activation [51]. ABCA1 plays a role in cardiovascular disease onset, and it is also correlated with impairments in insulin/glucose uptake metabolic processes when unregulated [48,49,50]. ABCA1’s expression was also shown to be involved in the bioavailability of dietary components such as vitamin E, lutein, and zeaxanthin [52,53]. In diabetic rats, ABCA1 is overexpressed in the small intestine epithelial and inflammation-mediator cells [45], suggesting different roles in the inflammatory cascade. Regarding colorectal cancer, ABCA1 presents higher expression in the advanced phases of the disease, as the deregulation of cholesterol transport improves the growth of tumor cells and metastasis [54]. Various phytochemicals have been shown to induce MDR-related ABC transporter inhibition in cell line models of various cancer types, as described in Table 1 for P-gp, MRP1, and ABCG2, suggesting their potential for the development of new pharmacological approaches to chemotherapy.

In this review, we will emphasize the potential of natural products to reverse MDR specifically in colorectal cancer, as it will be described below.

## 3. Natural Products as Potential Candidates to Overcome Colorectal Cancer MDR

Representing 8% of all newly diagnosed cancer cases, colorectal cancer is the most common type of malignant tumor in the intestinal tract, with high lethality worldwide (the fourth deadliest cancer), with developed countries being the most affected [82,83,84]. The development of colorectal cancer is often connected partially with hereditary factors, but obesity, sedentarism, poor nutritional choices, and/or alcohol and tobacco consumption play a significant role in tumor onset [82,83,84]. Contributing to its lethality, MDR plays a significant role in the low success rate of chemotherapy in colorectal cancer, as 90% of patients with metastases face the failure of this treatment option due to resistance to chemotherapeutic drugs [85]. Along the intestinal tract, three of the ABC transporters described above are expressed in both the small intestine and the colon and have been highly correlated with MDR: P-gp, MRP1, and ABCG2 [9].

Among the potential options for MDR reversion, products of natural origin arise as an extensive library of compounds with countless possibilities for chemical structures and for which various anti-tumor activities have been described. Some natural products, such as medicinal plants, have been widely used throughout human history for medicinal purposes and thus have high acceptability. Chemotherapy drugs are often expensive, present undesired toxicity, and, as seen above, are subjected to MDR. In addition to the anti-tumor activities described for various phytochemicals and herbal extracts, their ability to inhibit ABC transporters and modulate MDR highlights these products as potential co-adjuvants for conventional cancer treatment [85,86].

Depending on the compound molecular structure, the MDR reversion mechanism can be dependent on direct inhibition of the ABC transporter, suppression of the transporter genes, and/or be accompanied by anti-tumor activity exerted on target pathways such as PI3K (phosphoinositide 3-kinase) or NF-kB (nuclear factor kappa-light-chain-enhancer of activated B cells) [87,88]. Thus, natural products used in the human diet and phytochemicals commonly found in food products or herbal extracts, commonly referred to as nutraceuticals, will deserve a higher focus in this review. Various food components have been studied for their interaction with P-gp, with different effects on the modulation of this transporter activity. While green tea, rosemary extract, orange extract, mint extract, and apricot extract inhibited P-gp activity, other foodstuffs, such as St. John’s wort or grapefruit extract, inducted the transporter activity [89]. In this review, we focused on natural product research applied to the intestinal tract and more specifically to colorectal cancer. In this respect, a methanolic extract of *Momordica charantia* L. (bitter melon) was tested in HT-29 cells (human colorectal adenocarcinoma cell line) [90], results showed that *M. charantia* extract (100 µg/mL) induced a 10-fold decrease in doxorubicin’s IC_50_ when HT-29 cells were co-incubated with extract and doxorubicin, and the extract also sensitized HT-29 cells to doxorubicin when the cells were pre-exposed with extract (25 µg/mL) prior to doxorubicin exposure, being the effect related to the extracts ability to inhibit efflux transporters involved in MDR [90]. On the other hand, HT-29 cells displayed less sensitivity to the extracts when pretreated with doxorubicin [90]. *Sabiosa atropurpurea* L. (mourning bride) methanolic extracts, rich in various glycosidic derivatives of apigenin, luteolin, and quercetin, have also been shown to improve doxorubicin toxicity in Caco-2 cells (colorectal adenocarcinoma) as a result of inhibition of P-gp and MRP1 [91].

The effect of rosemary (*Rosmarinus officinalis* Spenn.) extracts, obtained by using a super-critical fluid pilot plant, were evaluated in SW620 (Dukes’ type C colorectal adenocarcinoma), SW620-5-FU-R (SW620 cells resistant to 5-fluorouracil; resistance generated by stepwise increases of 5-fluorouracil (up to 3 µM) over 15 months), and DLD-1 (Dukes’ type C colorectal adenocarcinoma) cells by González-Vallinas, et al. 2013 [92], who showed that, in addition to the anti-tumor activity of extracts, a synergistic effect was observed when cells were treated with extracts together with 5-fluoroacil, and the 5-fluoroacil-resistant SW620 cells were sensitized to the chemotherapeutic drug after being subject to different concentrations of extract (0–120 μg/mL; over 72 h). In this study, the mechanism of action of rosemary extracts was reported to be the inhibition of thymidine kinase, an enzyme responsible for the recovery of thymidylate synthetase, the target of 5-fluorouacil, with the putative effect on P-gp also suggested [92].

Regarding the positive or negative modulatory effect of extracts and their components over P-gp, a study using *Hypericum perforatum* (Saint John’s wort) methanol extract (300 µg/mL) and one of its main components, hypericin (3 µM), showed induction of P-gp expression in LS180 cells (Dukes’ type B colorectal adenocarcinoma), while in Caco-2 cells P-gp activity was mildly inhibited [93], suggesting that the modulatory effect is dependent on the used cell model. As seen in Table 2, most of the studies published are based on in vitro assays using well-characterized colorectal cancer cell models. Nevertheless, these cell cultures may vary in the expression of various transporters (not only ABC transporters), but they also may present different mutations that induce variations in key metabolic pathways. For example, Caco-2 cells do not present known mutations in several key pathways connected to the anti-tumor activity, such as the PI3K/AKT/mTOR or Ras/Raf/MAPK pathways, in addition to presenting a typical enterocyte differentiation [94]. Other colorectal cells, such as HT-29, present mutations in BRAF and in PI3K; DLD-1 and HCT116 cells present mutations in KRAS and PI3K; and SW620 cells are known for a mutation in the KRAS pathway [94].

**Figure 3:**
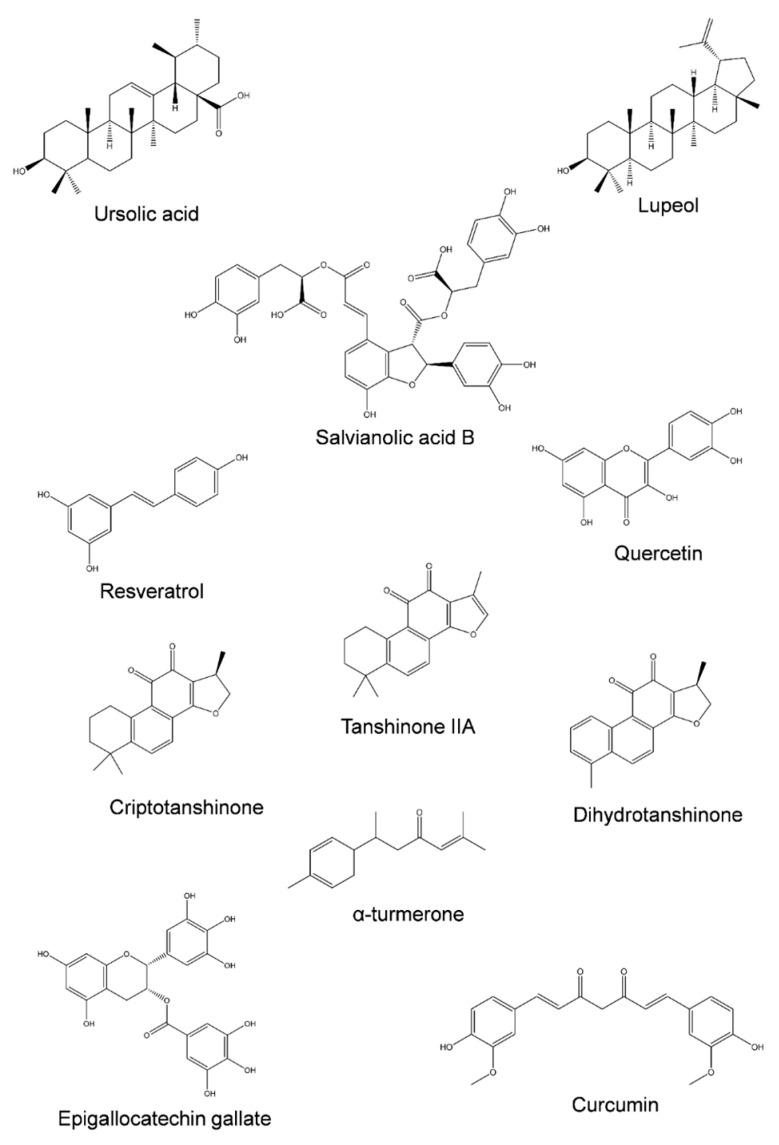
Structural diversity of phytochemicals capable of inhibiting ABC transporters involved in colorectal cancer MDR.

Concerning the natural compounds that are described to interfere with the re-regulation of MDR, Figure 3 presents the chemical structure of several phytochemicals capable of inhibiting MDR-related transporters in colorectal cancer cell lines, and a summary of their specific effects is listed in Table 2.

Concerning isolated phytochemicals, many have been proposed as co-adjuvants for chemotherapy drugs, for which their anti-tumor activity is also desirable. A promising case is ursolic acid (Figure 3). This pentacyclic triterpenoid is capable of inhibiting P-gp in various colorectal cancer cell line models (Table 2), which further supports the findings of the various studies [69,95,96]. In addition, the efflux of various P-gp substrates (5-fluoroacil, oxaliplatin, and doxorubicin) was inhibited by ursolic acid [69,95,96], showing the potential effect of this phytochemical as a co-adjuvant agent. Oleanolic acid, another pentacyclic triterpenoid, has also been shown to inhibit both P-gp and MRP1 [71]. Several plant species present both ursolic and oleanolic acids in their phytochemical compositions, such as plants from the *Thymus* genus (e.g., *Thymus carnosus* Boiss. [97], *Thymus pulegioides* L. [98], *Thymus serpyllum* L. [99], or *Thymus praecox* Opiz. [99]) and *Salvia* genus (e.g., *Salvia officinalis* L. [100], *Salvia sclarea* L. [101], or *Salvia aucheri* Benth [101]), that can be promising options for the formulation of co-adjuvants.

Similar findings were observed for lupeol, a pentacyclic triterpenoid whose target was the inhibition of ABCG2 [102]. As well for pentacyclic triterpenoids, the addition of a sugar moiety, such as saponin glycyrrhizic acid, that was shown to inhibit P-gp in Caco-2 cells [96], highlights the potential of these classes of compounds for such bioactivity.

A second class of compounds that have been highlighted for their ability to reverse MDR at the intestinal level are tanshinones [103,104], in particular tanshinone IIA, cryptotanshinone, and dihydrotanshinone (Table 2). These compounds are capable of inhibiting P-gp and are also commonly found in the *Salvia* genus [103,104]. Other classes of natural compounds, such as alkaloids obtained from natural products, have been reviewed for their capacity to reverse MDR [105].

However, polyphenolic compounds arise as a class within phytochemicals, with a large number of potential inhibitors of ABC transporters but also presenting inducers and subtractors of these proteins. As seen in Table 2, compounds such as salvianolic acid B, resveratrol, quercetin, or epigallocatechin gallate have been reported as inhibitors of MDR-related proteins. Salvianolic acid B is an inhibitor of both P-gp and ABCG2 [106,107]. The resveratrol effect in MDR reversion is performed through P-gp downregulation and suppression of its gene, regulated through the AMPK (AMP-activated protein kinase) pathway [108]. Rosmarinic acid (at 30 µg/mL) was found to downregulate the expression of both P-gp and ABCG2 in Caco-2 cells [109]. However, in HepG2 (hepatocarcinoma) cells, rosmarinic acid was shown to induce the expression of P-gp and ABCG2 [110], corroborating the idea of cell-specific effects. Rosmarinic acid was shown to increase the transport of two fluorescent probes (Rho123 and Ho33342) from the apical to the basolateral side while reducing the transport in the opposite direction [109], providing information on the complexity of the mechanism involving the action of phytochemicals on intestinal barrier transporters. Within phenolic compounds, the flavonoids class presents various compounds that have been screened for their interactions with ABC-transporters in several cell lines, not only from the intestinal tract. The flavonoids that reduce P-gp activity and have been reported for aglycones include quercetin, epigallocatechin gallate, epicatechin gallate, catechin gallate, hesperetin, and naringenin [87,111]. The inhibitory effect of these compounds is mostly related to their interaction with the substrate binding site by competitive inhibition, modulation of ATPase activity and ATP consumption, decrease in protein expression, or even their interaction with the lipidic bilayer, affecting the structural integrity of transporters [87,111]. On the other hand, flavonoids such as kaempferol or galangin seem to stimulate P-gp [111]. Concerning MRP1, quercetin, luteolin, kaempferol, apigenin, phloretin, naringenin, or chrysoeriol have been listed as MRP1 inhibitors, and ABCG2 activity can be reduced or inhibited by quercetin, hesperetin, or chrysin [87,111]. Nevertheless, a study performed in HCT-15 cells (Dukes’ type C colorectal adenocarcinoma) has shown that quercetin and kaempferol stimulate adriamycin efflux through P-gp, thus potentiating the transporter activity [112]. Other studies reported that P-gp expression in Caco-2 cells was increased after a four-week exposure to 10 µM of myricetin, epigallocatechin gallate, or quercetin, among others, as evaluated by the increase in P-gp mRNA expression [113]. While contradictory, it is plausible that flavonoid concentration, experimental conditions, or the cell line model chosen may contribute to different experimental outcomes. Even more, although this requires a significant comprehension of the modulation of ABC transporters by phytochemicals, it must be taken into account that P-gp, MRP1, and ABCG2 physiological functions are the efflux of xenobiotics, preventing their intracellular accumulation. Having this in mind, the ingestion of flavonoids may contribute to enhancing the intestinal barrier defenses against exogenous toxicants. A second remark is related to the use of herbal extracts; each extract presents a unique phytochemical profile with different ratios of each component. Adding to the complexity, flavonoids are often found as glycosidic derivatives and not as aglycones; usually the aglycones are the compounds tested. The balance between substrates, inhibitors, and inducers of ABC transporters in each plant extract may produce a different outcome.

**Table 2:** Natural compound effects on reversing chemotherapeutic drug resistance in colorectal cancer, using in vitro and in vivo models.

Compound	Experimental Model	Concentration	Effect	Ref.
Ursolic acid	HCT-8 and SW620 cells	20 µM	Increased effect of oxaliplatin; Reduced P-gp expression	[69]
	RKO, LoVo, and SW480 cells	20 µM	Reduced P-gp expression; Increased the sensitivity to 5-fluroacil and oxaliplatin	[95]
	Caco-2 cells	>200 µM	Chemosensitizing effect for doxorubicin; P-gp inhibition at high concentrations	[96]
Salvianolic acid B	HCT-8 cells	20 µg/mL	Increased sensitivity to 5-fluorouracil, cisplatin, vincristine and paclitaxel; Reduced P-gp expression	[107]
	LoVo and HCT-116 cancer stem cells xenografts in mice	0.36 g	Reduced ABCG2 expression	[106]
α-turmerone	Caco-2 cells	50 µg/mL	Reduced P-gp expression	[114]
Curcumin	HCT-8 and HCT-8/5-FU cells	5.5 µg/mL	Increased sensitivity to 5-fluorouracil; Reduced P-gp expression	[115]
	HCT-8 and HCT-8/5-FU cells	12.96 µg/mL	Increased 5-fluorouracil effect; Reduced P-gp expression	[116]
	SW620 and SW620/Ad300 cells	5.5 µM	Increased doxorubicin effect; Reduced P-gp activity	[117]
	In situ Cancerous Colon Perfusion Rat Model	50 mg/kg	Reduced P-gp expression	[118]
Quercetin	SW620/Ad300 cells	33 µM	Increased doxorubicin effect; Inhibited P-gp activity	[119]
Tanshinone IIA	SCID mice with Colo205 cell xenograft	20 mg/kg	Increased 5-fluorouracil effect; Downregulation of P-gp	[104]
Cryptotanshinone	Caco-2 and SW620/Ad300 cells	25 µM	Increased doxorubicin and irinotecan toxicity; P-gp transport inhibition	[103]
Dihydrotanshinone	Caco-2 and SW620/Ad300 cells	25 µM	Increased doxorubicin and irinotecan toxicity; P-gp transport inhibition	[103]
Cinobufagin	LoVo/ADR, HCT-116/L-OHP, and Caco-2/ADR cells	20 nM	Inhibited P-gp activity	[120]
Resveratrol	HCT-116/L-OHP cells	50 µM	Downregulation of P-gp	[108]
Epigallocatechin gallate	HCT-116 and DLD-1 cells	50 µM	Increased 5-fluorouracil effect; Suppressed *MDR1* expression	[59]
Lupeol	LoVo cells	50 µM	Increased oxaliplatin effect; ABCG2 suppression	[102]

Notes: HTC-8 cells: colorectal adenocarcinoma; SW620 cells: Dukes’ type C colorectal adenocarcinoma; SW480 cells: Dukes’ type B colorectal adenocarcinoma; LoVo cells: Dukes’ type C colorectal adenocarcinoma; Caco-2 cells: colorectal adenocarcinoma; HCT-116: colorectal carcinoma; RKO: colon carcinoma; DLD: Dukes’ type C colorectal adenocarcinoma; SW620/Ad300: P-gp overexpressing SW620 cells; HCT/5-FU: 5-fluoroacil-resistant HCT-8; LoVo/ADR and Caco-2/ADR: LoVo and Caco-2 cells with induced MDR and P-gp overexpression; HCT-116/L-OHP: oxaliplatin-resistant HCT-116 cells.

Additionally, the various phytochemicals present in an extract or in a natural product may present synergistic activities or antagonistic effects among themselves. Therefore, there is still a need for further studies concerning the modulation of ABC transporters by phytochemicals to clarify which ones are suitable as co-adjuvants and also concerning the role of the patient’s diet in the interaction between therapeutic drugs and the intestinal barrier, the ultimate goal being a balance between xenobiotic export for homeostasis maintenance and drug uptake and bioaccumulation into cancer cells for optimal therapeutic purposes.

The only natural compound, not obtained from plants, presented in Table 2 is cinobufagin. This bufanolide steroid, obtained from *Bufo gargarizans* (Asiatic toad) skin and auricular gland, is one of the main components of traditional Chinese medicine [120]. It was observed that this steroid induced non-competitive inhibition of P-gp in LoVo, Caco-2, and HCT-116 cells overexpressing P-gp, at very low concentrations (20 nM) [120]. A similar activity was described for another component used in Chinese traditional medicine, bufalin, a steroid obtained from Chinese toad’s venom [121]. Bufalin, at 20 nM, was also able to inhibit P-gp, reversing the sensitivity to chemotherapy drugs in P-gp-overexpressing cell line models of colorectal cancer (HCT-8 and LoVo) [121]. It is equally relevant to evaluate MDR reversal in colorectal cancer by other phytochemicals, namely those that induce inhibition of efflux transporters in other cancer types but have not yet been assessed for colorectal cancer. As cells are equipped with different transporters, one should not assume that the bioactivity of a certain compound is equally exerted on other cell lines, such as colorectal cancer cells.

In cells other than those of the colon, many studies are of interest. For example, 6-gingerol, a phenolic compound obtained from ginger, produces anti-tumor activity towards human hepatocarcinoma cells (HepG2 and Huh7) and a human cervix adenocarcinoma (HeLa) [122]. In hepatocarcinoma cells, 6-gingerol was shown to increase doxorubicin cytotoxicity [122]. In KB-C2 cells (human endocervical adenocarcinoma), daunorubicin bioaccumulation was increased in cells exposed to 6-gingerol [73]. As doxorubicin and daunorubicin are P-gp substrates, it is hypothesized in both studies that 6-gingerol may be exerting this effect through P-gp inhibition [73,122]. The anti-tumor effect of 6-gingerol was also reported for HCT-166, a colorectal cancer cell line model, with a low IC_50_ (1.5 µM) [122]. However, in Caco-2 cells exposed to this phytochemical, there was no increase in doxorubicin bioaccumulation [122]. In addition, a different study also confirmed that 6-gingerol is not a P-gp substrate in Caco-2 cells [123] and thus may not actively interact with P-gp in this cell line.

However, in addition to the limitations of drug bioavailability, those of phytochemical bioavailability must also be addressed. Due to the uncertainty that still exists regarding phytochemical absorption and metabolization, which affect their bioavailability, their application in pharmaceutical formulations cannot be linear and dependent solely on the results obtained in in vitro assays [124]. In the case of intestinal tract and colorectal cancer treatment, these factors present a lesser limitation, as both drugs and phytochemicals may interact with the target site without being absorbed into the systemic circulation [125]. As described above, some phytochemicals act as ABC-transporters but may also be substrates. Ursolic acid, for example, is known to inhibit P-gp activity in an interaction that is likely dependent on the terpenoid’s hydrophobicity and should increase its affinity for the transporter [73]. However, ursolic acid also increased P-gp’s ATPase activity, which indicates that the phytochemical is also a substrate, and thus the inhibition is performed through competitive inhibition, limiting drug efflux [73] and also limiting ursolic acid’s bioavailability. This is one aspect of a major concern in the use of natural products: the herb-drug or phytochemical-drug interaction. Several natural products have been shown to negatively interact with common drugs, producing adverse symptoms [126]. The safety of coadministration of drugs and natural products (such as medicinal and aromatic plants) must be accessed before engaging in vivo studies, and it should also be taken into account in real-world cases in which patients are also undergoing complementary medicine [127,128,129].

Among the interactions that may improve or impair the chemotherapeutic activity of drugs is the up-regulation of receptors such as the pregnane-X-receptor (PXR), liver-X-receptor (LXR), retinoid-X-receptor (RXR), farnesoid-X-receptor (FXR), aryl hydrocarbon receptor (AhR), or constitutive androstane receptor (CAR), some of which are reported to increase the expression of ABC transporters [130,131,132,133]. In addition to the up-regulation of some ABC transporters, some of these receptors can also induce the expression of enzymes responsible for drug metabolization, such as cytochrome P450 (CYP)3A4 [131,134]. In HepG2 cells, various plant extracts were shown to increase PXR-mediated *CYP3A4* gene induction [135]. A second study reported that in a study comprising 123 plant extracts, 16% of the samples were shown to strongly induce PXR activation and 14.63% to strongly induce AhR activation; however, the inhibition of both CYP enzymes and P-gp was also produced by various extracts [131], revealing the variation of effects induced by herb-drug interactions in the potential efficacy of chemotherapy drugs and new pharmaceutical products. Particularly in colorectal cancer, Harmsen, et al. 2010 [136] reported that the resistance to several chemotherapy drugs was partly induced by increased P-gp induction in response to PXR activation [136]. Among common natural products used in diet or traditional medicine, the activities of these receptors, ABC transporters, and detoxication enzymes are heterogeneous. There are reports of various products such as green tea, ginseng, turmeric, or St. John’s wort, for example, with studies reporting P-gp induction, CYP enzyme activation/inhibition, and the potential involvement of receptors such as PXR [137]. Epicatechin, for example, has been shown to target and activate mouse PXR in skeletal muscle [134].

With the currently available information, these factors influence the validity of the inclusion of natural compounds in pharmacological formulations for chemotherapy. Advances in these fields of study are still dependent on the clarification of the mechanism of action behind phytochemicals’ activity and phytochemical-drug interaction, without which the safety and stability of new formulations cannot be assured. Nevertheless, some formulations have been successful in the various steps of scientific validation being proposed for clinical trials. According to ClinicalTrials.gov, a database provided by the United States National Library of Medicine, several phytochemicals described in this review have been used in clinical trials aimed at colorectal cancer prevention and treatment. Among them, the safety of resveratrol formulations was assessed in patients with colorectal cancer metastasis [138] and in patients with tumors that could be surgically removed [139]. EGCG (94%; purified from green tea extract) is also under evaluation for its chemopreventive activity in resectable colorectal cancer cases [140] and is the major component of green tea extracts, which are also being used in clinical trial studies [141,142]. The chemopreventive potential of curcumin [143,144], as well as that of more complex matrices such as ginger root extracts [145] or, in the case of pomegranate extracts, the effect of their consumption after diagnosis, were also under study [146].

Despite the existence of various clinical trials and the potential described for natural products, the lack of knowledge regarding the core cellular processes involved in their health-promoting effects is still a reality, which may be limiting the exploitation of these natural resources by the pharmaceutical industry. In addition, the advance of nanomedicine must be taken into account, as nanotechnology may offer a solution to improve both drug and phytochemical availability at the target site as well as potentially prevent unwanted interactions [147,148]. It is necessary to better understand the basic mechanisms of chemoprevention and anti-tumor activities of these phytochemicals and their application in colorectal cancer in vitro in order to transition to in vivo studies and potential pharmaceutical applications.

## 4. Conclusions

Colorectal cancer is among the cancer types with higher lethality, partly due to the lack of treatment options, where chemotherapy is largely affected by multidrug resistance. As a hotspot of import and export of endogenous compounds and xenobiotics, ABC transporters are essential to maintaining the intestinal barrier’s homeostasis. ABC transporters are the major intervenients in xenobiotic defense mechanisms, a role that occasionally counters the therapeutic approach to colorectal cancer. Either as a part of food components ingested regularly or as potential co-adjuvants for chemotherapy, natural products and phytochemicals in particular have been for long studied for their anti-tumor activities, for which the ability to regulate drug efflux and increase their effectiveness is a highly sought-after bioactivity. Several phytochemicals and other natural compounds have been reported as inhibitors of P-gp, MRP1, and ABCG2, the major targets in MDR at the intestinal level. This is a route of opportunity for new pharmaceutical formulations with lower drug doses, lower toxicity to normal tissues, and higher efficacy, aiming to increase chemotherapy success and improve the outcomes of colorectal cancer. Nevertheless, not all natural compounds are ABC transporter inhibitors. Especially in complex matrices such as herbal extracts, the existence of inhibitors, stimulators, and/or substrates must be taken into account, as well as how the diet of a patient may affect the success of the chemotherapy. Despite the modulatory effect promoted by phytochemicals, these do not replace chemotherapy; however, knowing their modulatory effect, they may be included in pharmaceutical formulations for chemotherapy. On the other hand, knowledge regarding the mechanism of action of phytochemicals on ABC transporters, as well as phytochemicals’ distribution in food products, can help create treatment-facilitating diets that indicate the foods to eat and avoid. Phytochemicals are regarded as nutraceutical molecules that contribute to overall homeostasis; thus, equilibrated and phytochemical-rich diets present a support for the prevention and treatment of several diseases.

## Figures and Tables

**Table 1:** Phytochemicals targeting P-gp, MRP1, and ABCG2 in in vitro models and their potential for MDR reversal.

Phytochemical	MDR-Related Transporter Inhibition			Ref.
	P-gp	MRP1	ABCG2	
Epigallocatechin gallate	Caco-2 CH^R^C5 KB-C2 MCF-7 HepG2	A549 DLD-1 HL-60 NB4	MCF-7	[55,56,57,58,59,60,61]
Quercetin	Caco-2 MCF-7	S*f*9 inside-out vesicles	HEK293 S*f*9 inside-out vesicles	[62,63,64,65,66]
Ursolic acid	HCT-8 SW480 KB-C2		HEK293 Ovarian CSCs MDCK	[47,66,67,68,69]
Oleanolic acid		Ma104 SW982 SK-UT-1	HEK293	[47,70,71]
Kaempferol			HEK293 MDCK	[47,72]
Gingerol	KB-C2	PC-3		[73,74]
Curcumin	Caco-2 SiHa	SiHa MCF-7 MDA-MB-23	MCF-7 MDA-MB-23	[75,76,77]
Resveratrol	KB-C2 Caco-2 rat everted gut sac model	Caco-2 AML-2	Pancreatic CSC Caco-2	[78,79,80,81]

Notes: CH^R^C5 cells: Chinese hamster ovary resistant; Caco-2 cells: human colorectal adenocarcinoma; KB-C2 cells: human endocervical adenocarcinoma; HepG2 cells: human hepatocarcinoma; A549 cells: human lung carcinoma; DLD-1 cells: human colorectal adenocarcinoma; NB4 cells: acute promyelocytic leukemia; HL-60 cells: human acute myeloid leukemia: MDCK cells: Madin-Darby canine kidney; HEK293 cells: human embryonic kidney; Ma104 cells: monkey kidney embryo; SW982 cells: human synovial sarcoma; SK-UT-1 cells: human leiomyosarcoma; HCT-8 cells: human colorectal adenocarcinoma; SW480 cells: human colorectal adenocarcinoma; PC-3 cells: human prostate carcinoma; MDA-MB-23: SiHa cells: human squamous cell carcinoma: AML-2 cells: human acute myeloid leukemia; CSC: cancer stem cells.

## Data Availability

Not applicable.
